# Bevacizumab-induced arrhythmia in a patient with lung adenocarcinoma: A case report

**DOI:** 10.1097/MD.0000000000034799

**Published:** 2023-09-01

**Authors:** Jing Li, Furong Zhang, Yuanyuan Lu

**Affiliations:** a Department of Integrated Traditional Chinese and Western Medicine, Tongji Hospital, Tongji Medical College, Huazhong University of Science and Technology, Wuhan, China; b Department of Pharmacy, Maternal and Child Health Hospital of Hubei Province, Tongji Medical College, Huazhong University of Science and Technology, Wuhan, China.

**Keywords:** adverse reaction, arrhythmia, bevacizumab, case report, lung adenocarcinoma

## Abstract

**Rationale:**

Cardiotoxicity is an important side effect of vascular endothelial growth factor inhibitors therapy in the treatment of cancer. Massive studies have shown bevacizumab-related hypertension, venous, and arterial thrombosis.

**Patient concerns:**

A 56-year-old female patient was treated with bevacizumab monotherapy for lung adenocarcinoma. The patient was detected a poor R-wave increase with slight ST segment elevation in V1–V3 leads, and ventricular arrhythmia.

**Diagnosis:**

The incidental arrhythmia caused by bevacizumab was considered.

**Interventions:**

The patients received aspirin and amiodarone (0.2 g tid) to treat arrhythmia. After consultation with the cardiology department, the patient received a diagnostic coronary angiography. Coronary angiography showed 30% of the right coronary artery stenoses and no obvious organic stenosis in the left main artery, left anterior ascending, or left circumflex.

**Outcomes:**

The patient exhibited disappearance of chest tightness and rapid heartbeat after the treatment of amiodarone. Electrocardiogram monitoring results returning to normal.

**Lessons:**

This is the first reported case of bevacizumab-associated arrhythmia. It is advisable to consider the risk of arrhythmia in bevacizumab monotherapy or combines treatment.

## 1. Introduction

Bevacizumab is one of the first targeted therapies and the first approved angiogenesis inhibitor. As a recombinant antibody against vascular endothelial growth factor (VEGF), bevacizumab is used for the treatment of various cancers, including colorectal cancer, non-small cell lung cancer, glioblastoma, cervical carcinoma, and ovarian cancer.^[1]^ It has been reported that VEGF inhibitors treatment is associated with important cardiovascular adverse events such as systemic hypertension, congestive heart failure, corrected QT prolongation, and venous and arterial thrombosis.^[2]^ In this case report we describe the case of a female patient with arrhythmia during bevacizumab therapy for lung adenocarcinoma. This is the first report of bevacizumab monotherapy related arrhythmia.

## 2. Case presentation

The present study reports on a case of lung adenocarcinoma in a 56-year-old female patient. The patient had been diagnosed with lung adenocarcinoma and epidermal growth factor receptor gene mutation in December 2019. She was administered Icotinib combined with bevacizumab for targeted therapy. This time the patient was admitted for the next cycle of bevacizumab intravenous injection. The patient had no history of hypertension, mental illness, cardiovascular or cerebrovascular disease except for 10 years of routine insulin treatment for diabetes.

An initial evaluation of the patient suggested well with a temperature of 36.7°C, blood pressure of 111/66 mm Hg, a pulse of 60 times/minute, and a respiration rate of 20 per minute. She had a normal heart rhythm and no obvious murmur was heard in the auscultation area of each valve.

On the fifth day after the injection of 300 mg bevacizumab, the ambulatory electrocardiographic monitoring of the patient was abnormal. The average heart rate of the patient was 64 beats per minute (bpm) with a maximum heart rate of 99 bpm and a minimum heart rate of 46 bpm. Among the 24-hour electrocardiogram (ECG) recordings, atrial premature beats were found 9 times and one run of 4 consecutive beats of atrial tachycardia with an atrial rate of 106 beats per minute. The patient had 31 times premature ventricular beats and 4 runs of ventricular tachycardia (4–10 consecutive beats and the rate of ventricular tachycardia range 53–168/minute) during 24 hours. The “R-on-T” phenomenon was detected as ventricular velocity frequency reaches 168 times/min. The ECG showed a poor R-wave increase with slight ST segment elevation in V1–V3 leads. ST-T segment changed in the inferior wall, anterior wall, and anterior lateral walls leads during rapid ventricular rate. T wave changes could be seen in part of the time when the patient had chest tightness, rapid heartbeat, and other uncomfortable symptoms. Heart-rate variability analysis showed the values of standard deviation of normal to normal interval (155 ms) and SD of the averages of NN intervals (146 ms) in the normal range.

## 3. Treatment

The patients received aspirin and amiodarone (0.2 g tid) to treat arrhythmia. After consultation with the cardiology department, the patient received a diagnostic coronary angiography. Coronary angiography showed 30% of the right coronary artery stenoses and no obvious organic stenosis in the left main artery, left anterior ascending, or left circumflex. The examination results ruled out coronary artery disease. Cardiac color Doppler ultrasound and myocardial infarction laboratory investigations results showed normal.

The patient exhibited disappearance of chest tightness and rapid heartbeat after the treatment of amiodarone. ECG monitoring results returning to normal.

## 4. Discussion

Cancer therapy-induced cardiovascular toxicity is a growing clinical problem. The cardiotoxicity rates range from 0.06% to 1.14% in patients undergoing antineoplastic drug treatment.^[3]^ Although VEGF inhibitors have become one of the most promising strategies in cancer therapy, cardiovascular safety has been the most challenging aspect of anti-VEGF/VEGF receptor agent development and therapy. VEGF blockade leads to endothelial dysfunction, which reduces nitric oxide formation in endothelial cells and then leads to impaired vasorelaxation function. Inhibition of the nitric oxide signal increases platelet aggregation and adhesion, leading to thrombosis. These factors result in the development of hypertension, cardiovascular, and cerebrovascular ischemia, and increased the risk of bleeding. The risk of cardiotoxicity in patients receiving VEGF inhibitors is 7.4% for hypertension, 1.8% for thromboembolism, 1.7% for cardiac ischemia, and 2.3% for general cardiac dysfunction.^[4]^

Bevacizumab specifically binds to the VEGF-A protein, thereby inhibiting the process of endothelial cell proliferation and neovascularization. Among numerous studies of bevacizumab-related cardiovascular adverse events, hypertension and vascular thromboembolism are the most widely reported. The incidence of heart failure is less reported in monotherapy of bevacizumab, and as high as 14% in combination therapy.^[5]^ A phase II study reported a very high incidence of 20% of sinus tachycardia and supraventricular tachycardia in bevacizumab combined with everolimus treatment for refractory metastatic renal cell carcinoma.^[6]^ However, there are no reported cases of arrhythmia or ventricular tachycardia due to bevacizumab monotherapy.

In our case, the patient was found a series of ECG abnormalities such as ST-segment elevation and R-on-T phenomenon. ST-segment elevation in leads V1 through V3 is a marker for sudden death in patients without demonstrable structural heart disease. It is considered to reflect acute transmural ischemia caused by an occlusion of an epicardial coronary artery by a blood clot.^[7]^ The “R-on-T” phenomenon was detected when the ventricular velocity frequency of the patient reaches 168 times/min. The “R-on-T phenomenon” is the superimposition of an ectopic beat on the T wave of a preceding beat. Early observations suggested that R-on-T was likely to initiate sustained ventricular tachyarrhythmias. Subsequent studies found that R-on-T hardly produces malignant ventricular arrhythmias in normal hearts. However, the R-on-T ventricular premature beat may be of importance in subsets of patients at risk for polymorphic ventricular tachycardia or ventricular fibrillation, such as those with acute myocardial ischemia, the Brugada syndrome, the malignant form of early repolarization, and idiopathic ventricular fibrillation.^[8]^ Luckily, coronary angiography and cardiac color doppler ultrasound results ruled out a coronary artery or heart diseases. The incidental arrhythmia of the patient was most likely caused by bevacizumab.

## 5. Conclusion

In the case of a patient receiving bevacizumab administration, the risk of arrhythmia should be considered, and regardless of the type of cancer or whether combines treatment, careful administration of bevacizumab is necessary.

## Author contributions

**Methodology:** Jing Li, Furong Zhang

**Writing – original draft:** Jing Li, Furong Zhang

**Supervision:** yuanyuan Lu
